# Transporter and metabolizer gene polymorphisms affect fluoroquinolone pharmacokinetic parameters

**DOI:** 10.3389/fphar.2022.1063413

**Published:** 2022-12-12

**Authors:** Nurul Annisa, Melisa I. Barliana, Prayudi Santoso, Rovina Ruslami

**Affiliations:** ^1^ Department of Biological Pharmacy, Biotechnology Pharmacy Laboratory, Faculty of Pharmacy, Universitas Padjadjaran, Sumedang, Indonesia; ^2^ Unit of Clinical Pharmacy and Community, Faculty of Pharmacy, Universitas Mulawarman, Samarinda, Indonesia; ^3^ Center of Excellence for Pharmaceutical Care Innovation, Universitas Padjadjaran, Sumedang, Indonesia; ^4^ Division of Respirology and Critical Care, Department of Internal Medicine, Faculty of Medicine, Universitas Padjadjaran-Hasan Sadikin Hospital, Bandung, Indonesia; ^5^ Division of Pharmacology and Therapy, Department of Biomedical Sciences, Faculty of Medicine, Universitas Padjadjaran, Bandung, Indonesia

**Keywords:** pharmacogenetics, *UGT1A1*, *UGT1A9*, *SLCO1B1*, *ABCB1*, moxifloxacin, sitafloxacin, trovafloxacin

## Abstract

Tuberculosis (TB) is an infectious disease that occurs globally. Treatment of TB has been hindered by problems with multidrug-resistant strains (MDR-TB). Fluoroquinolones are one of the main drugs used for the treatment of MDR-TB. The success of therapy can be influenced by genetic factors and their impact on pharmacokinetic parameters. This review was conducted by searching the PubMed database with keywords polymorphism and fluoroquinolones. The presence of gene polymorphisms, including *UGT1A1*, *UGT1A9*, *SLCO1B1*, and *ABCB1*, can affect fluoroquinolones pharmacokinetic parameters such as area under the curve (AUC), creatinine clearance (C_Cr_), maximum plasma concentration (C_max_), half-life (t_1/2_) and peak time (t_max_) of fluoroquinolones.

## Introduction

Multidrug Resistant-Tuberculosis (MDR-TB) is a severe problem in various parts of the world, and Tuberculosis (TB) cases are particularly prevalent in India and Indonesia (WHO, 2020a). However, the use of pharmacogenomic and pharmacokinetic aspects as therapeutic parameters were expected to have a positive impact on treatment and may achieve an 80% reduction in TB incidence rates by 2030 as described in the WHO End TB strategy.

Drug responses of individual patients can be determined by the drug pharmacokinetic parameters, and these responses can be affected by single nucleotide polymorphisms (SNPs) in genes that encode drug-metabolizing enzymes and transporters; the influence of these SNPs on drug response is called pharmacogenetics. Therefore, comprehensive molecular understanding and clinical information for precise treatment of individuals are needed to improve the outcome therapy (Roden and Tyndale, 2011). Genetic polymorphism is due to naturally existing variants in genes that occur in more than 1% of the population. Polymorphism may influence the action of a drug by changing the pharmacokinetic or pharmacodynamic profile (Belle and Singh, 2008).

Transport proteins or transporters are membrane channels and molecular pumps that facilitate the movement of ions, small molecules, macromolecules, and drugs across membranes (Nelson et al., 2008). The movement of biochemical compounds through biological membranes is critical to the absorption, distribution, metabolism, and excretion of nutrients, neurotransmitters, and drugs (Overington et al., 2006; Ware, 2006; Giacomini et al., 2010; Yan, 2010; Rask-Andersen et al., 2011). The dynamic partnerships of transporters with other signaling molecules in subcellular locations are regarded as essential processes for cellular function. The attenuation of transporter gene sequence by polymorphisms often contributes to complex human diseases and individual drug responses (Ware, 2006; Cardoso et al., 2010; Yan, 2010; Longo et al., 2011; Rask-Andersen et al., 2011; Ueda, 2011).

In shorter MDR-TB treatment regimens, fluoroquinolones such as levofloxacin (L-isomer ofloxacin) and moxifloxacin (8-methoxy fluoroquinolone) are an important class of drugs (group A) used in the initial treatment phase for 4–6 months and the continuation phase for 5 months (WHO, 2020b). Fluoroquinolones such as levofloxacin and moxifloxacin are effective against gram-positive and gram-negative anaerobic bacteria through bactericidal action that acts *via* inhibition of the topoisomerase II (DNA gyrase) and topoisomerase IV enzymes required for bacterial DNA replication, transcription, repair, and recombination (FDA, 2008; Fàbrega et al., 2009; FDA, 2016).

Fluoroquinolone antibiotics play a significant role in the elimination of bacteria in the treatment of infections, necessitating accuracy in drug administration to maintain their efficacy and safety, which can be influenced by several circumstances. Therefore, the objective of this study was to determine explicitly how human genetic variation can alter the pharmacokinetic profile of fluoroquinolone antibiotic and how it also impact in clinical implication.

## Materials and methods

This narrative review used articles published in PubMed obtained using the combination of “polymorphism” OR “single nucleotide polymorphism” OR “SNP” AND “fluoroquinolone” AND “pharmacokinetic” as keywords and found 387 articles. Finally, 6 out of 387 studies were reviewed to identify gene polymorphisms and their effect on the pharmacokinetic parameters of fluoroquinolones. Due to the limitations of published studies, we also searched several other studies that have been performed on other drugs for more comprehensive approaches to gene polymorphisms.

## Result and discussion

Metabolic processes in the liver play an important role in influencing drug levels in the body. Drug metabolism in the liver can result in the formation of drugs that are more hydrophilic and that are then excreted through the liver, kidneys, and/or intestines. Drug metabolism involves the chemical biotransformation of drug molecules by enzymes present in the body. In addition, drug transporters facilitate the movement of drugs and metabolites in and out of cells and organs (Taxax and Bhartam, 2014).

Drug metabolism pathways consist of phases I and II and may include phase III. The phase I pathway is generally controlled by the Cytochrome P450 (CYP450) family, the main group of enzymes that chemically modify drugs so that they are more soluble in water and are then easily excreted by the kidneys and/or liver. The phase II pathway of drugs/metabolites involves enzymatic conjugation with endogenous hydrophilic compounds assisted by transferase enzymes; phase II metabolic enzymes are UDP-glucuronosyltransferase (UGT), Sulfotransferases, N-acyl transferases, Glutathione S-transferases, N-acetyl transferases, and Methyl transferases (Taxax and Bhartam, 2014). Phase III pathways are classified into two main superfamilies: ATP-binding cassette (ABC) proteins and solute carrier (SLC) transporters. The phase III pathway is facilitated by drug transporters that in general are transmembrane proteins that facilitate the transport of large molecules and/or ionized molecules into or out of the cell. ABC transporters require energy (ATP) to actively absorb or efflux the drug from one side of the cell membrane to the other, whereas SLCs enable the passage of certain solutes (e.g., sugars and amino acids) across the membrane while actively transporting other solutes despite their electrochemical gradients by coupling the process with another solute or ion (Almazroo et al., 2017). Fifty-two percent of moxifloxacin’s oral or intravenous dose is metabolized by glucuronide and sulfate conjugation (phase II metabolism). While the CYP450 system is not involved in the metabolic process of the moxifloxacin (FDA, 2016).

The pharmacokinetic profile of a drug can be influenced by internal factors that cannot be modified, such as genetic components, and several genes are known to be involved in the pharmacokinetic profile of fluoroquinolones. We found the study in fluoroquinolones antibiotics group are moxifloxacin, sitafloxacin, and trovafloxacin as fourth-generation fluoroquinolones. The gene found were uridine 5′-diphospho-glucuronosyltransferase family 1 member A1 (*UGT1A1*), 5′-diphospho-glucuronosyltransferase family 1 member A9 (*UGT1A9*)*,* solute carrier organic anion transporter family member 1B1 (*SLCO1B1*), and ATP-binding cassette subfamily B member 1 (*ABCB1*) may affect fluoroquinolones pharmacokinetic parameters, including creatinine clearance (C_Cr_), area under the curve (AUC), maximum plasma concentration (C_max_), half-life (t_1/2_) and time to the maximum plasma concentration (t_max_).

**FIGURE 1:**
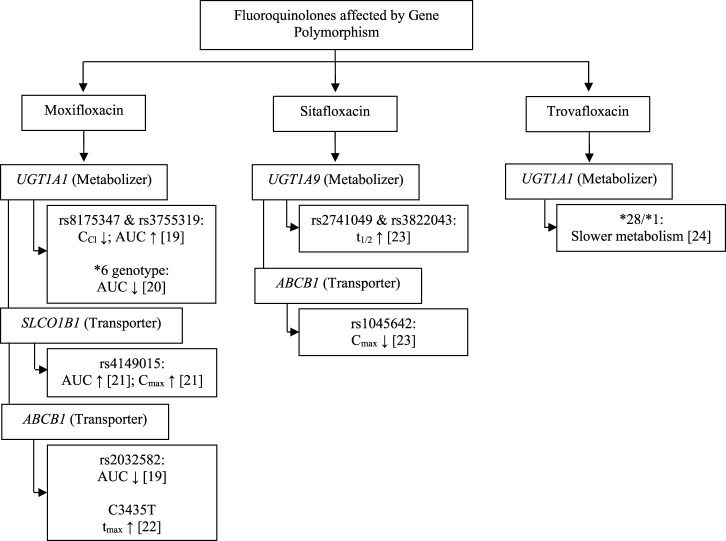
Fluoroquinolones pharmacokinetic parameter affected by gene polymorphisms.

Thus, the pharmacokinetic profile of fluoroquinolones may be affected by proteins expressed by the *UGT1A1*, *UGT1A9*, *SLCO1B1*, and *ABCB1* genes (Figure 1; Tables 1, 2). UGT functions as a drug metabolizer, while the roles of SLC and ABC as transporters will certainly affect the pharmacokinetic profile. The metabolism of moxifloxacin by UGT with SNPs at rs8175347 and rs3755319 reduces C_Cr_ and increases AUC in healthy subjects, in contrast to studies that show a reduction in AUC with the UGT1A1*6 genotype. *SLCO1B1* encodes organic anion transporting polypeptide 1B1 (OATP1B1), which acts as a moxifloxacin drug transporter. The rs4149015 SNP of *SLCO1B1* increases the AUC and C_max_ of moxifloxacin, whereas the p-glycoprotein drug transporter encoded by *ABCB1* decreases the AUC, C_max_, and increases T_max_. Studies have shown a similar decrease in drug exposure and a prolonged time to reach the peak drug level in the body (Weiner et al., 2007; Hasunuma et al., 2016; Naidoo et al., 2018; Weiner et al., 2018).

**TABLE 1:** The effect of transporter and metabolizer human gene polymorphism on moxifloxacin pharmacokinetic parameter.

Gene	Genotyping method	Polymorphism	Study population		Discussion	References
			Number	Ethnic		
*UGT1A1*	Taqman^®^ Genotyping OpenArray™	rs8175347	230	African	Association between gene polymorphism and moxifloxacin pharmacokinetics (reducing C_Cr_ and increasing AUC)	Naidoo et al. (2018)
		rs3755319				
	TaqMan SNP Genotyping Assay, Life Techologies Japan	*6	79	Japanese, Chinese, Korean and Caucasian	*6 carriers had a significantly lower AUC_inf_ of M2 moxifloxacin compared with the other genotypes	Hasunuma et al. (2016)
		Genotype				
*SLCO1B1*	Allelic Discrimination Assay (TaqMan C_32325356), Thermo Fisher Scientific	rs4149015	49	African and American	The median moxifloxacin AUC_0–24_ was 46% higher and the median C_max_ was 30% higher in 4 (8%) participants who had the SLCO1B1 g.-11187 AG genotype	Weiner et al. (2018)
		g.-11187G>A				
*ABCB1*	Taqman^®^ Genotyping OpenArray™	rs2032582	230	African	The area under the concentration-time curve from 0 to 24 h (AUC_0–24_) for moxifloxacin decreased 27%	Naidoo et al. (2018)
	TaqMan Real Time PCR	C3435T	16	Black, White non-Hispanic, White Hispanic, Asian	Significant increase in peak time (T_max_) in subjects with MDR1 3435CC compared to other genotypes	Weiner et al. (2007)

**TABLE 2:** The effect of transporter and metabolizer human gene polymorphism on sitafloxacin and trovafloxacin pharmacokinetic parameter.

Gene	Genotyping method	Polymorphism	Study population		Discussion	References
			Number	Ethnic		
*ABCB1*	Sanger Sequencing, Shanghai Sangon Bio-Tech Co., Ltd.	rs1045642 (sitafloxacin)	30	Chinese	Had a significant effect on the C_max_ dose sitafloxacin	Sun et al. (2021)
*UGT1A9*		rs2741049 (sitafloxacin)			Had a significant effect on the sitafloxacin t_1/2_	
		rs3822043 (sitafloxacin)			Had a significant effect on the sitafloxacin t_1/2_	
*UGT1A1*	Enzyme assay: UGT Isoforms-expressing Systems	*28/*1 (trovafloxacin)	Human liver microsome	The trovafloxacin glucuronidation in liver microsomes from UGT1A1*28/*28 carriers was significantly slower than that in microsomes from UGT1A1*1/*1		Fujiwara et al. (2015)

Polymorphism of *UGT1A1*, *UGT1A9*, and *ABCB1* also affects the pharmacokinetic profile in sitafloxacin and trovafloxacin (Figure 1; Table 2). A study on sitafloxacin showed that there was an association of genetic polymorphism of the human drug transporter *ABCB1* rs1045642 in subjects with heterozygous or homozygous genotype variants with a lower C_max_ of sitafloxacin than that in subjects without variants (*p* < 0.05) (Sun et al., 2021). SNPs in *UGT1A9* rs2741049 and rs3832043 have a longer half-life (t_1/2_) than those in subjects without the variant; this was thought to occur due to decreased metabolism and disposition of sitafloxacin. However, this study did not show significant changes in AUC in either the *ABCB1* or *UGT1A9* groups (Sun et al., 2021). Acyl glucuronidation of trovafloxacin has been studied in human liver microsomes and also in UGT isoform-expressing systems in human liver microsomes. UGT1A1*28/*28 carriers were significantly slower metabolism than that in microsomes from UGT1A1*1/*1, suggesting that *UGT1A1* is the main contributor to the glucuronidation of trovafloxacin (Fujiwara et al., 2015). At the molecular level, an influx transporter implicated in the membrane transport of quinolone antibacterial drugs (levofloxacin) has been identified for the first time as a result of the current work, in conclusion. OATP1A2, the discovered transporter molecule, is expressed in several tissues, including the small intestine, blood-brain barrier, liver, lung, and testis. As a result, it may play a part in regulating the intestinal absorption, tissue distribution, and hepatic excretion of these substances (Maeda et al., 2007).

Covariates can have an impact on the pharmacokinetic profile of fluoroquinolones in addition to genetic variables. According to a study conducted in Africa, gender, height, and body size (fat-free mass) may affect the pharmacokinetic characteristics of moxifloxacin, but these variables were adjusted before the pharmacokinetic analysis (Naidoo et al., 2018). Studies on the body weight, body mass index (BMI), and C_Cr_ of subjects of Japanese, Chinese, Korean, and Caucasian ancestry have been adjusted for pharmacokinetic moxifloxacin outcomes at a body weight of 70 kg (Hasunuma et al., 2016). Age, race, and weight were all correlated with geographic origin (African and American). The moxifloxacin AUC_0-24_ and C_max_ were significantly increased by the moxifloxacin milligrams per kilogram dosage and genotype of variant g.11187G.A in the *SLCO1B1* gene (rs4149015), but not by geographic location (Weiner et al., 2018). To adjust factors that can impact the pharmacokinetic profile of moxifloxacin, values from monitored univariate tests, sex, ethnicity (Black, White non-Hispanic, White Hispanic, and Asian), and univariate test results were employed (Weiner et al., 2007). Sitafloxacin can reduce C_max_ by 50% when consumed by subjects who are fasting or having high fat foods intake, but it does not affect t_max_, t_1/2_ elimination, or total exposure (AUC_0-t_ and AUC_0-∞_). Despite only being possible in one dosing group of 10 participants, it is possible to affect the pharmacokinetic properties of ciprofloxacin, including t_1/2_ and C_max_ (Sun et al., 2021).

### UGT1A1 and UGT1A9

*UGT1A1* is a member of the UGT family and encodes a UDP-glucuronosyltransferase, an enzyme of the glucuronidation pathway (Genecards, 2022), (National Library of Medicines, 2022). This enzyme catalyzes glucuronidation during phase II of drug metabolism, especially in conjugate reactions (Guillemette et al., 2000; Balram et al., 2002; Zhang et al., 2007). Moxifloxacin and trovafloxacin are metabolized *via* glucuronide and sulfate conjugation by glucuronosyltransferase and sulfotransferase (Moise et al., 2000), (Vincent et al., 1998). Therefore, genetic variations of *UGT1A1* may affect the pharmacokinetic profile and clinical response of moxifloxacin and trovafloxacin.

Studies in South African patients (of black African ethnicity) with TB (Naidoo et al., 2018), *UGT1A1* rs8175347, and rs3755319 were significantly associated with the alteration of pharmacokinetic parameters for moxifloxacin. After controlling for other factors, it was shown that having the TA 5/6 repeat in rs8175347 was linked to a 20.6% poorer clearance and roughly a 26% higher AUC (*p* = 0.001). Subjects with the AC and AA rs3755319 genotypes had 11.6% higher clearance than those with the CC genotype in the model (*p* = 0.032) (Naidoo et al., 2018). Other studies showed that UGT1A1*6 (211G > A; G71R) carriers had a significantly lower total exposure to the drug (AUC_inf_) for the glucuronide conjugate (M2 metabolite) moxifloxacin than that in the other genotypes (*p* < 0.0001), although the metabolism of moxifloxacin itself was not influenced by variation of the *UGT1A1* genotype. There were significant differences between Japanese, Chinese, Korean, and Caucasian populations in C_max_, AUC_inf_, and C_Cr_ of moxifloxacin. The average AUC_inf_ and C_max_ compared to the parent compound in this study showed significant differences between the Japanese and Korean groups. AUC_inf_ M2 in Caucasian races showed a higher value than in East Asian population groups (Japanese, Chinese and Korean) and was associated with differences in the frequency of UGT1A1*6 genotypes. These studies showed that ethnic differences may affect the pharmacokinetic parameters of drugs (Hasunuma et al., 2016). In a study using human liver microsomes, trovafloxacin glucuronidation was substantially slower in liver microsomes from UGT1A1*28/*28 carriers than it was in *UGT1A*1*1/*1 carriers (Fujiwara et al., 2015). According to these findings, UGT1A1*28/*28, rs8175347, and rs3755319 carriers have a poor metabolizer phenotype, while carriers of the *6 genotype have a hyper metabolizer phenotype (Naidoo et al., 2018), (Hasunuma et al., 2016), (Fujiwara et al., 2015).

The genetic variations of *UGT1A1* also affected the pharmacokinetic profile of other drugs, such as telmisartan, irinotecan, dolutegravir, letermovir, and axitinib (Table 3). In general, the effect of the *UGT1A1* polymorphism on other drugs also has an influence on the pharmacokinetic profile (AUC and C_Cr_) and moxifloxacin.

**TABLE 3:** *UGT1A1* gene polymorphism on telmisartan, irinotecan, dolutegavir, latermovir, axitinib pharmacokinetic parameter.

Polymorphism	Genotyping method	Study population			Drug	Disussion	References
		Number	Ethnic	Condition			
rs4124874	Matrix-Assisted Laser Disorption/Ionization Time-of Flight Mass Spectrometry	58	Chinese	Hypertension	Telmisartan	Affected telmisartan bioavailability, Lower creatinine clearance and higher bioavailability in female with CC and CA genotype (high triglyceride)	Huang et al. (2019)
*28	Real-Time Allelic Discrimination PCR Assays on a DNA Engine Chromo4 System Bio-Rad Lab, United States)	93	European	HIV	Dolutegravir	Homozygosity was associated with a 79% increase in AUC_0-24_ (*p* = 0.001; 27% if analyzed individually	Elliot et al. (2020)
rs4148323	BioProcessing Solutions Alliance in Piscat-away, New Jersey	296	Asian, Black, White & Other	Prevention CMV Infection	Letermovir	An allele was present predominantly in Asian participants and was associated with an increase in AUC compared with non-carriers	Kobie et al. (2019)
*6 and *28	Pyrosequencing or Direct Sequencing	176	Japanese	Cancer	Irinotecan	Significantly reduce AUC ratios	Minami et al. (2007)
*6 221GA (rs4148323)	Tm Analysis Using a Quenching Probe	46	Japanese	Cancer	Axitinib	C_0_ dan AUC_0-12_ in patients with *UGT1A1* poor metabolizer were significantly higher than those in patients with *UGT1A1* extensive metabolizers (polymorphism were significantly associated with the plasma axitinib level)	Igarashi et al. (2018)
*27 686CA (rs35350960)							

A study on the hypertension drug telmisartan showed that there was a genotype difference (rs4124874) associated with decreased clearance and increased bioavailability, and studies of dolutegravir and letermovir showed similar results with an increase in AUC (Huang et al., 2019; Kobie et al., 2019; Elliot et al., 2020). Studies on irinotecan (UGT1A1*6 and *8) and axitinib (UGT1A1*6 221GA and *27 686CA) showed a reduction in AUC (Minami et al., 2007), (Igarashi et al., 2018).

Similarly with *UGT1A1*, *UGT1A9* also encodes a UDP-glucuronosyltransferase, an enzyme of the glucuronidation pathway that transforms small lipophilic molecules. UDP-glucuronosyltransferases (UGT) as a catalyst for the phase II biotransformation reaction in a lipophilic substrate conjugated with glucuronic acid to increase the polarity of the metabolite, which in turn can facilitate excretion in urine or bile. In addition, it has an important role in the elimination of drugs, xenobiotics, and endogenous compounds (Gene Cards, 2022a). Studies on sitafloxacin at rs2741049 and rs3832043 showed a significant effect on longer drug half-life t_1/2_ (*p* < 0.05). Inferring a poor metabolizer, rs2741049 and rs3822043 may play a part in the pharmacokinetic profile of the drug (Sun et al., 2021), another study at rs3832043 showed a possible effect on acetaminophen metabolism in neonates (Linakis et al., 2018). Thus, *UGT1A9* may have a role in the pharmacokinetic profile of the drug.

It can be summarized the effect of *UGT1A1* and *UGT1A9* can affect metabolic processes that have an impact on decreasing C_Cr_, decreasing t_1/2,_ and increasing AUC in the fluoroquinolone antibiotics (moxifloxacin, sitafloxacin, and trovafloxacin) (Naidoo et al., 2018), (Sun et al., 2021), (Fujiwara et al., 2015). An increase in AUC needs to be considered to avoid drug side effects and can be considered in treatment interventions such as lowering drug doses for the safety of drug use and the effectiveness of therapy.

### SLCO1B1

The *SLCO1B1* gene is located on chromosome 12p12.1, 796 base pairs, and is an important pharmacokinetic gene. The function of the OATP1B1 protein, encoded by *SLCO1B1*, is to enable the transport of several compounds (hormones, toxins, and drugs) from the blood to the liver for elimination (Genetic Home Reference, 2022), (Ensembl Asia, 2022). OATP1B1 is located on the sinusoidal membrane of human hepatocytes, where it mediates the uptake of its substrates from portal blood into the hepatocytes (Takaaki et al., 1999), (Oshiro et al., 2010).

4 (8%) persons with the *SLCO1B1* g.11187 AG genotype had a median moxifloxacin AUC_0-24_ that was 46% higher and a median C_max_ that was 30% higher than 45 participants with the wild-type GG genotype (median AUC0–24 from the model [*p* 0.005, ANCOVA]; median C_max_ from the model [*p* 0.009, ANCOVA]) (Weiner et al., 2018). This suggests that subject with g.-11187G>A shows poor metabolizer phenotype. Elevated levels of moxifloxacin can lead to risk for blood and lymphatic system orders (anemia), gastrointestinal disorders (nausea, diarrhea, vomiting, constipation, abdominal pain, dyspepsia), metabolic and nutritional disorders (hypokalemia), nervous system disorders (headache, dizziness) and QT prolongation (FDA, 2016). Increased AUC and C_max_ need to be seen to avoid drug side effects and can be considered in treatment interventions such as lowering doses for the safety of drug use.

SNPs of *SLCO1B1* may affect the pharmacokinetic profiles of other drugs (Table 4). Studies on atorvastatin polymorphisms in (SLCO1B1*15) and fluvastatin (c.521TA>G) showed an AUC-enhancing effect. Similarly, studies on methotrexate (388A>G and wild-type (AA) for 388A>G) showed the largest decrease in clearance in this genotype (Lee et al., 2010; Hirvensalo et al., 2019; Schulte et al., 2021). Conversely, studies on rifampin (c.388AA) and repaglinide/nateglinide (SLCO1B1*1B/*1B) in the Finnish ethnic group showed a decrease in drug concentrations in the body. In addition, the same study on repaglinide (SLCO1B1*1B/*1B) in ethnic Chinese was consistent with previous studies showing decreased AUC (Kalliokoski et al., 2008; He et al., 2011; Dompreh et al., 2018). Several studies also showed an effect of increasing the AUC that was similar to that of moxifloxacin.

**TABLE 4:** *SLCO1B1* gene polymorphism on atorvastatin, 2-hydroatorvastatin, rifampin, repaglinide, nateglinide, methotrexate, fluvastatin & repaglinide pharmacokinetic parameter.

Polymorphism	Genotyping method	Study population			Drug	Discussion	References
		Number	Ethnic	Condition			
*15 for c.388A > G	Direct Sequencing, Using an Automated Genetic Analyzer	290	Korean	Heathy subject	Atorvastatin and 2-Hydroxyatorvastatin	SLCO1B1*15 allele increased the AUC of atorvastatin	Lee et al. (2010)
388A>G (rs2306283)	VANTAGE Using custom Designed Multiplexed MassARRAY IPLEX Gold SNP Paels, Evaluated in a MassArray Typer 4.0	106	American	Lymphoblastic Lymphoma	Methotrexate	388A>G and 521T>C affect methotrexate clearance variability	Schulte et al. (2021)
521T>C (rs4149056)							
c.521T>C (rs4149056)	TaqMan Assays on QuantStudio 12Kflex Real-Time PCR System	200	Finnish	Healthy subject	Fluvastatin	c.521T>C has an enantiospecific effect on active 3R,5S-fluvastatin increased AUC.	Hirvensalo et al. (2019)
c.388AA	Taqman Genotyping on ViiA 7 Real-Time PCR	113	Ghanaian	Tuberculosis	Rifampin	c.388AA genotype (found in 2 children) was associated with low rifampin concentration compared with c.388GG.	Dompreh et al. (2018)
c.463AA							
*1A/*1B	TaqMan Allelic Discrimination with Applied Biosystems 7300 Real-Time PCR System	16	Finnish	Heathy subject	Repaglinide & Nateglinide	The SLCO1B1*1B/*1B genotype is associated with reduced plasma concentrations of repaglinide, consistent with an enhanced hepatic uptake by OATP1B1, but has limited effects on the pharmacokinetics of nateglinide	Kalliokoski et al. (2008)
*1B/*1B	Polymerase Chain Reaction-Restriction Fragment Length Polymorphism (PCR-RFLP), with Little Modification	22	Chinese	Healthy subject	Repaglinide	SLCO1B1*1A/*1B or *1A/*1A genotype and SLCO1B1*15/*1A or *5/*1A genotype had significantly higher AUC_0-∞_ than participants with SLCO1B1*1B/*1B genotype	He et al. (2011)
*1A/*1B or *1A/*1A						There was a difference in clearance between the two genotype groups but it was not significant	

### ABCB1

*ABCB1* is a member of the ABC family. *ABCB1* is a member of the ABC family. ABC proteins are divided into seven subfamilies, namely, ATP binding cassette subfamily A (ABC1), ATP binding cassette subfamily B (MDR/TAP), ATP binding cassette subfamily C (ABCC), ATP binding cassette subfamily D (ABCD), ATP binding cassette subfamily E (ABCE), ATP binding cassette subfamily F (GCN20), and ATP binding cassette subfamily G (WHITE) (Gene Cards, 2022b), (Human Genome Organisation, 2022). ABC proteins are found on chromosome 7q21.12, 323 base pairs that span 209.6 kb with 29 exons (Gene Cards, 2022b). The p-glycoprotein encoded by *ABCB1* plays a key role in the elimination of drugs in the first pass of orally administered drugs, thereby limiting their bioavailability by excreting the drug through the epithelium that faces the lumen of the small intestine and colon and from the canaliculi facing the hepatic bile. The drug substrate will be removed from the systemic circulation through urine *via* the proximal renal tubule and through biliary excretion (Fromm, 2004). The expression and function of p-glycoprotein are *ABCB1* SNPs-dependent. Changes in p-glycoprotein expression and function will affect the absorption, tissue distribution, and excretion of drug substances. Therefore, transporters genetic variations potentially affected the fate of drugs as well as the effectiveness of therapy (Marzolini et al., 2004).

For the *ABCB1* SNP rs2032582, only one person in the cohort under study had the CA genotype. The patient with the CA genotype had a 40% lower prehepatic bioavailability and a comparable decrease in AUC when the effect of the rs2032582 SNP was taken into account in the population PK model (*p* = 0.01) (Naidoo et al., 2018). However, in another study, in univariate analyses of the pharmacogenetic data obtained with moxifloxacin plus rifampin, cases with the *MDR1* 3435CC genotype showed a significant increase in the time to the peak concentration of moxifloxacin (T_max_) compared to cases with the other genotypes, but there were no differences in the mean peak concentration of moxifloxacin [a 23% lower geometric mean for the 3435 CC genotype (*p* = 0.08)] (Weiner et al., 2007).

Two studies showed in-line results in the ABCB1 gene. A decrease in AUC and an increase in T_max_ occurred with moxifloxacin, whereas in sitafloxacin there was a decrease in C_max_ (*p* < 0.05) (Naidoo et al., 2018), (Weiner et al., 2007), (Sun et al., 2021). According to the moxifloxacin pharmacokinetics results, *ABCB1* rs2032582 and C3435T suggest a hyper metabolizer phenotype, while rs1045642 on sitafloxacin suggests a poor metabolizer. Low levels of drugs in the blood in the use of antibiotics will risk the occurrence of drug resistance and failed treatment. This data can be taken into consideration in treatment interventions such as increasing or adjusting the drug dose until it reaches the expected level.

Genetic variations of *ABCB1* may also affect the pharmacokinetic profiles of other drugs (Table 5). *ABCB1* polymorphism studies have been performed using other drugs and show that lower drug levels are in line with those used for moxifloxacin and sitafloxacin (decreased C_max_). Studies on other drugs showed similar results where variations in pharmacokinetic parameters tended to decrease the blood levels of the drug. Studies on aripiprazole showed that the 1236TT genotype compared with the CC genotype had a lower clearance of aripiprazole, and also lower AUC and C_max_ of dehydro-aripiprazole (it is an active metabolite). The azithromycin study showed that individuals with a heterozygous genotype of 2677GT/3435CC could inhibit intermediate levels of AUC and C_max_. In the sunitinib study, the mutant genotype (CT/TT) had a greater Cl/F compared with that of the wild genotype (CC). Clopidogrel (C3435) and tacrolimus (3435T) studies have revealed decreased plasma drug levels (Belmonte et al., 2018; Nazir et al., 2020; Woo et al., 2016; Wang et al., 2015; Naito et al., 2015).

**TABLE 5:** *ABCB1* gene polymorphism on aripiprazole, azithromycin, sunitinib, clopidogrel, and tacrolimus pharmacokinetic parameter.

Polymorphism	Genotyping method	Study population			Drug	Discussion	References
		Number	Ethnic	Condition			
1236TT	Real_Time Polymerase Chain Reaction (PCR)	148	Spanish	Healthy subject	Aripiprazole	1236TT had lower clearance of aripiprazole (*p* = 0.023) and AUC (*p* = 0.039) and C_max_ of dehydro-aripiprazole (*p* = 0.036) compared to C/C	Belmonte et al. (2018)
2677GG/3435CC	Polymerase Chain Reaction-Restriction Fragment Length Polymorphism (PCR-RFLP)	16	Pakistani	Healthy subject	Azithromycin	C_max_ was significantly higher in 2677GG/3435CC as compared to 2677GT/3435CT and 2677TT/3435TT (p-value = 0.02)	Nazir et al. (2020)
2677GT/3435CT							
2677TT/3435TT							
C3435T, rs1045642	High-Performance Liquid Chromatography (HPLC)	31	Asian	Metastatic renal cell carcinoma	Sunitinib	Mutant genotype CT/TT) on *Cl*/*F* of sunitinib was higher than 31.14% (*p* = 0.006) as compared with the wild genotype (CC)	Woo et al. (2016)
C3435T, rs1045642	Seqnom MassArray Technology (San Diego, United States)	401	Chinese	Acute Coronary Syndrome	Clopidogrel	The carriers of C3435T were associated with lower levels of plasma clopidogrel and its active (clopi-H4) and inactive (CLPM) metabolites (all *p* = 0.05 vs non-carriers)	Wang et al. (2015)
3435 T	Polymerase Chain Reaction-Restriction Fragment Length Polymorphism (PCR-RFLP)	70	Japanese	Rheumatoid Arthritis	Tacrolimus	The 3435TT group had higher dose-normalized blood concentrations of tacrolimus and 13-O-demethylate	Naito et al. (2015)

### Clinical implications

Treatment of MDR-TB is critical to eradicating *tuberculosis*. Genetic variations (SNPs) in genes that encode drug metabolizers or transporter proteins may affect the response to treatment. Identification of these SNPs is expected to provide specific information on the alteration of pharmacokinetic profiles, especially that of fluoroquinolones, a key drug in MDR-TB therapy (group A). Therefore, dose adjustments for the phenotype that appears (hyper metabolizer or poor metabolizer) may be required. In addition, it is also possible to consider changing the drug, because in antibiotic therapy a certain dose must be reached for efficacy without causing toxic effects. Therefore, it is deemed necessary to carry out pharmacogenetic studies to see the genetic profile of a population. Hence, genetic profile information can be used as a database for genetic screening for personalized treatment recommendations.

### Limitation of the review

A limitation of this review is that studies on relevant SNPs and their effect on the pharmacokinetic profile of fluoroquinolones are limited. Hence, the number of subjects in those included studies are also limited and this may not be representative enough for the population. We also discovered that SNPs variation in the same gene can influence the pharmacokinetic profile of other drugs. This information also enriched the description of altered functions of transporters (*ABCB1 and SLCO1B1*) and metabolizers (*UGT1A1 and UGT1A9*) although their effect on fluoroquinolones still needs further study.

## Conclusion

SNP polymorphisms that have been known to affect the pharmacokinetic parameters of fluoroquinolone as an important drug in the treatment of MDR-TB are found in the *UGT1A1*, *UGT1A9*, *SLCO1B1*, and *ABCB1* genes. *UGT1A1* and *UGT1A9*, genes that encode enzyme metabolizers for fluoroquinolones, can reduce C_Cr,_ t_1/2_, and influence AUC. *SLCO1B1*, a gene that encodes the OATP1B1 protein as a drug transporter for moxifloxacin, can increase the AUC and C_max_. *ABCB1*, a gene that encodes p-glycoprotein as a drug transporter for fluoroquinolones, has the effect of decreasing AUC and C_max_, and increasing T_max_.
